# Arbuscular Mycorrhizal Symbiosis Mitigates Iron (Fe)-Deficiency Retardation in Alfalfa (*Medicago sativa* L.) Through the Enhancement of Fe Accumulation and Sulfur-Assisted Antioxidant Defense

**DOI:** 10.3390/ijms21062219

**Published:** 2020-03-23

**Authors:** Md. Atikur Rahman, Monika Parvin, Urmi Das, Esrat Jahan Ela, Sang-Hoon Lee, Ki-Won Lee, Ahmad Humayan Kabir

**Affiliations:** 1 Grassland and Forage Division, National Institute of Animal Science, Rural Development Administration, Cheonan 31000, Korea; atikbt@korea.kr (M.A.R.); sanghoon@korea.kr (S.-H.L.); 2 Molecular Plant Physiology Laboratory, Department of Botany, University of Rajshahi, Rajshahi 6205, Bangladesh; monikaparvin1333@gmail.com (M.P.); dasurmi2911@gmail.com (U.D.); ahmad.kabir@ru.ac.bd (A.H.K.)

**Keywords:** arbuscular mycorrhizal fungi, symbiosis, Fe-deficiency, alfalfa, *Medicago sativa*, Fe-mobilization, Fe-chelate reductase

## Abstract

Iron (Fe)-deficiency is one of the major constraints affecting growth, yield and nutritional quality in plants. This study was performed to elucidate how arbuscular mycorrhizal fungi (AMF) alleviate Fe-deficiency retardation in alfalfa (*Medicago sativa* L.). AMF supplementation improved plant biomass, chlorophyll score, Fv/Fm (quantum efficiency of photosystem II), and Pi_ABS (photosynthesis performance index), and reduced cell death, electrolyte leakage, and hydrogen peroxide accumulation in alfalfa. Moreover, AMF enhanced ferric chelate reductase activity as well as Fe, Zn, S and P in alfalfa under Fe-deficiency. Although Fe-transporters (*MsIRT1* and *MsNramp1*) did not induce in root but *MsFRO1* significantly induced by AMF under Fe deficiency in roots, suggesting that AMF-mediated Fe enhancement is related to the bioavailability of Fe at rhizosphere/root apoplast rather than the upregulation of Fe transporters under Fe deficiency in alfalfa. Several S-transporters (*MsSULTR1;1*, *MsSULTR1;2*, *MsSULTR1;3,* and *MsSULTR3;1*) markedly increased following AMF supplementation with or without Fe-deficiency alfalfa. Our study further suggests that Fe uptake system is independently influenced by AMF regardless of the S status in alfalfa. However, the increase of S in alfalfa is correlated with the elevation of GR and S-metabolites (glutathione and cysteine) associated with antioxidant defense under Fe deficiency.

## 1. Introduction

Iron (Fe) deficiency is a major nutritional limitation for crops development. Several conditions, such as high soil pH, the presence of bicarbonate in soil, abundant of ferric Fe^3+^ and geological factors, are linked to Fe unavailability in soil [1,2]. Fe deficiency causes less Fe accumulation in plants leading to severe chlorosis, poor growth, and maturation in plants [3,4]. Beside this, several co-factors containing Fe are involved with cellular processes, such as mitochondrial respiration, the formation of protein structure and photosynthesis in plants [5]. Alfalfa (*Medicago sativa*), a strategy-I plant possess reduction-based mechanisms in which ferric chelate reductase activity (FRO) and Fe-regulated transporter (*IRT1*, *NRAMP1*, etc.) play an essential role in Fe uptake [6,7]. In addition, the induction of plasma membrane H^+^-ATPase and/or acidification enhances the mobilization of Fe under deficiency in plants [8,9]. Along with the transporter genes, organic acids [10] and sulfur metabolites [11] may also facilitate Fe uptake in plants.

Arbuscular mycorrhizal fungi (AMF), mainly belonging to the Glomeromycota phylum, establish mutualistic communication with vascular plants [12]. AMF improved the nutritional status of plants primarily through the channel of fungal hyphae in response to different nutritional deficiencies [13,14]. Due to AMF symbiosis, uptake mechanisms of nutrient transporters are activated or constitutively-expressed [15,16]. Furthermore, the regulation of genes due to AMF might be associated with cytological and metabolic alternations in plants [17]. The effect of AMF on phosphorus uptake, mainly accomplished by symbiotic phosphate uptake pathway in plants, is well understood [18,19]. AMF symbiosis demonstrated to be beneficial for boosting Fe level in a few plant species under low Fe availability [4,20]. Studies also demonstrated that S-metabolites plays a vital role in improving the S status in plants due to AMF symbiosis [21]. Results also suggest that pathways linked to S metabolites are induced due to AMF, which in turn increases nutrient availability for plants [22]. However, the mechanism(s) and expression pattern of metal ion transporter due to AMF inoculation is still complicated to the readers. Along with the nutritional improvement, AMF also increases the activities of several enzymes associated with the accumulation of reactive oxygen species (ROS) induced by abiotic stresses in plants [23]. While this effect is evident, the background scenario of how AMF triggers antioxidant defense is largely unknown in plants under Fe-deficient conditions.

Alfalfa is a perennial legume mainly used as forage crop [24]. It also contributes to soil fertility because of its nitrogen fixing ability [24]. It is also a source of bio-fuel and is able to accumulate heavy metals [24,25]. Although Fe deficiency causes retardation in the growth of alfalfa, no extensive efforts have been made on the role of AMF to identify the mechanistic basis of improving Fe status. Therefore, the present study was designed whether or how AMF alleviates Fe deficiency retardation in alfalfa. Emphasis was given to the interactions of different minerals along with the transporter genes. We also investigated the effect of metabolites associated with AMF-mediated regulation of antioxidant defense in alfalfa under Fe deficiency.

## 2. Results

### 2.1. Impact of AMF on Alfalfa Morpho-Physiological Indices

**Figure 1:**
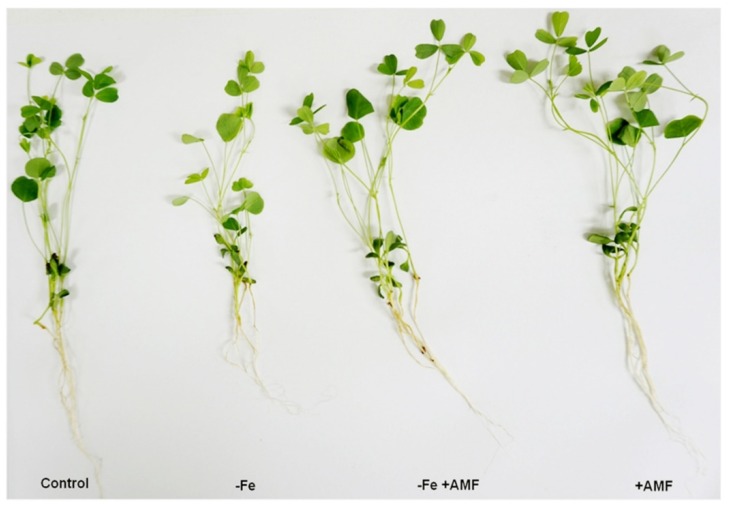
Changes of alfalfa phenotype. Fe deficiency (−Fe)-induced chlorosis, along with role of Arbuscular mycorrhizal fungi (AMF) to alleviate Fe deficiency retardation in alfalfa. Photograph of alfalfa plants were taken after 4 weeks of treatment.

**Figure 2:**
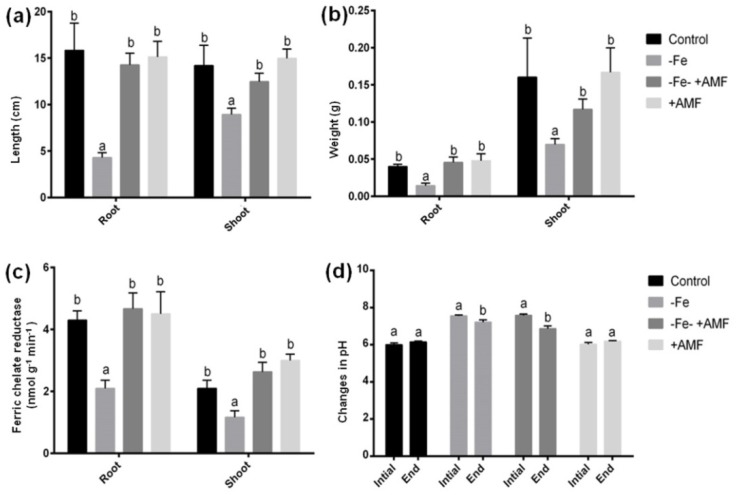
Effects of Fe deficiency and AMF on morpho-physiological parameters in alfalfa. (**a**) Root-shoot length, (**b**) root-shoot biomass, (**c**) ferric chelate reductase activity, and (**d**) changes of solution pH under Fe deficiency with or without AMF. Data represent means ± SD (*n* = 3). Different letters indicate significant difference at *p* < 0.05 level.

AMF supplementation showed colonization with clear hyphae and vesicle in the root of alfalfa plants (Supplementary Figure S1). In this study, Fe deficiency resulted in a significant change of the alfalfa phenotype along with the length and dry weight of both root and shoot compared to the non-treated controls (Figure 1 and Figure 2). Plant supplemented with AMF showed a significant increase in length as well as their root and shoot biomass compared to the plants cultivated under Fe deficiency (Figure 2a,b). Furthermore, ferric chelate reductase (FCR) activity significantly decreased following Fe deficiency in either root or shoot compared to non-treated controls. However, AMF supplied with or without Fe deficiency (+AMF, −Fe+AMF) showed a significant enhancement of ferric chelate reductase activity in both root and shoot compared to Fe-deficient (−Fe) plants (Figure 2c). In addition, the pH of the cultivation media measured at the beginning and the end of cultivation showed no significant changes. However, the bicarbonate induced Fe deficiency resulted in a significant decrease in pH level in the presence or absence of AMF, although the decrease was greater under AMF supplementation (Figure 2d).

**Figure 3:**
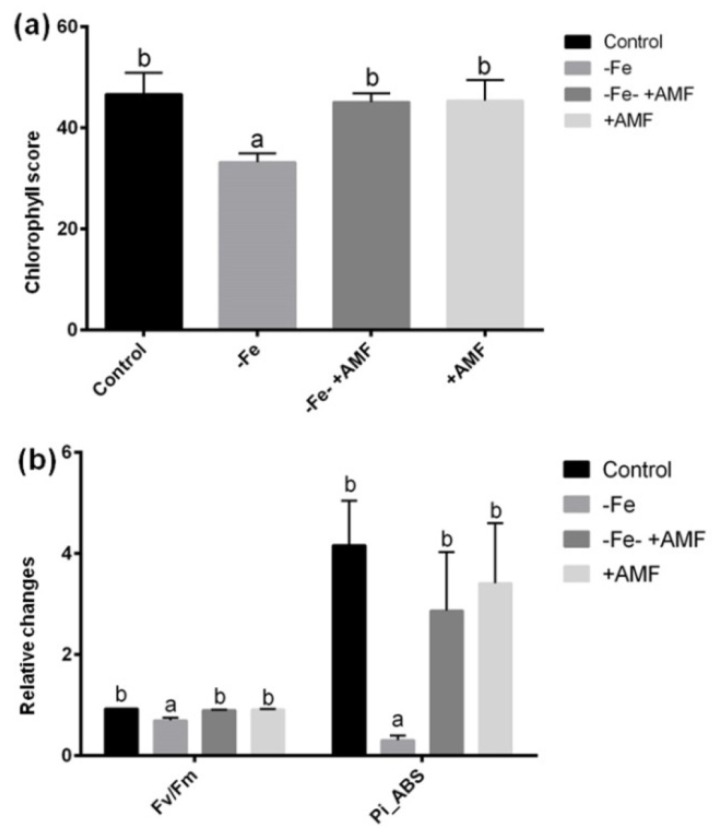
Effects of Fe deficiency and AMF on alfalfa photosynthetic indices. (**a**) Regulation of chlorophyll score, (**b**) Fv/Fm (quantum efficiency of photosystem II), and Pi_ABS (photosynthesis performance index) under Fe deficiency with or without AMF. Data represent means ± SD (*n* = 3). Different letters indicate significant difference at *p* < 0.05 level.

We measured SPAD and OJIB data to determine the effect of AMF on preventing photosynthesis damage caused by Fe deficiency. Fe deficiency caused a significant decline of chlorophyll score along with Fv/Fm (quantum efficiency of photosystem II) and Pi_ABS (photosynthesis performance index) due to Fe deficiency compared to controls that were restored in alfalfa plants in response to AMF addition (Figure 3a,b). Plants supplemented with AMF grown under control conditions similar to SPAD and Fv/Fm and Pi_ABS data to that of controls.

### 2.2. Impact of AMF on the Mineral Content 

ICP-MS analysis was performed in order to determine the concentration and interactions of major elements in alfalfa root and shoot. As expected, Fe deficiency resulted in a significant decrease in Fe, Zn, S, and Ca concentrations in both root and shoot of alfalfa plants compared to the non-treated controls (Table 1). The addition of AMF with Fe deficiency (−Fe + AMF) showed a significant increase in Fe, Zn, S, and Ca concentration in either root or shoot compared to Fe-deficient alfalfa plants. Plants grown with AMF showed similar Fe, Zn, S, and Ca concentrations in root and shoot to those plants non-treated control or grown under Fe deficiency with AMF supplementation (−Fe + AMF) (Table 1). Further, P concentration significantly decreased due to Fe deficiency in comparison with non-treated controls in both root and shoot (Table 1). The addition of AMF under Fe deficiency (−Fe + AMF) resulted in a significant increase in P concentration in either root or shoot compared to Fe-deficient alfalfa.

Moreover, plant grown with AMF supplementation (+AMF) showed similar enhancement of P concentration in root and shoot to that of non-treated controls, which are also significantly higher than the plants grown under Fe deficiency (−Fe) or Fe deficiency with AMF supplementation (−Fe + AMF) (Table 1). In this study, we did not find any significant change in K concentration in either root or shoot among the treatments (Table 1). Further, Fe deficiency showed no significant changes in Mg concentrations in root compared to control. However, the addition of AMF with or without Fe deficiency (+AMF, −Fe+AMF) resulted in a significant increase in Mg concentration in roots compared to the plants grown without AMF.

### 2.3. Role of AMF Overcoming Oxidative Stress in Alfalfa

**Figure 4:**
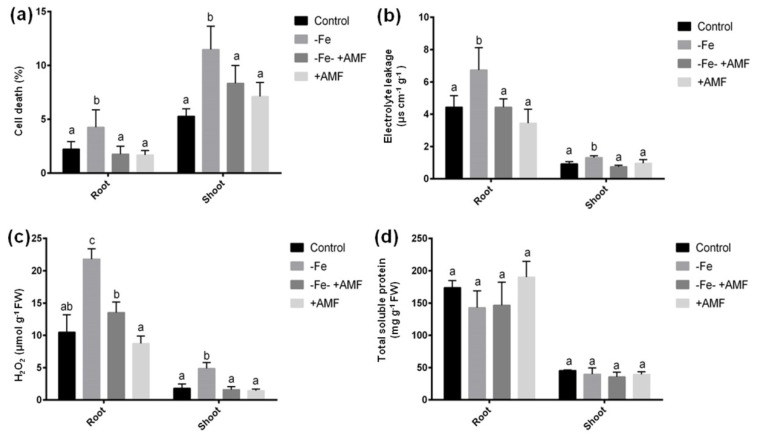
Effects of Fe deficiency and AMF on oxidative stress indicators in alfalfa. (**a**) Cell death, (**b**) electrolyte leakage, (**c**) H_2_O_2_ accumulation, and (**d**) changes of total soluble protein. Data represent means ± SD (*n* = 3). Different letters indicate significant difference at *p* < 0.05 level.

A number of stress indicators were checked to observe whether AMF mitigates the Fe-deficiency induced stresses in alfalfa. In this study, cell death (%), electrolyte leakage, and H_2_O_2_ concentration significantly enhanced in response to Fe deficiency in both root and shoot of alfalfa plants compared to controls (Figure 4a–c). However, the addition of AMF with or without Fe deficiency (+AMF, −Fe+AMF) resulted in a significant decrease in these biochemical stress indicators compared to Fe-stressed (−Fe) plants. Although the reduction of these stress indicators was greater in plants cultivated with AMF supplementation (+AMF). Total soluble protein content did not show any significant changes in either root or shoot among the treatments (Figure 4d).

### 2.4. Effect of AMF on the Expression of Fe and S Transporter Genes

**Figure 5:**
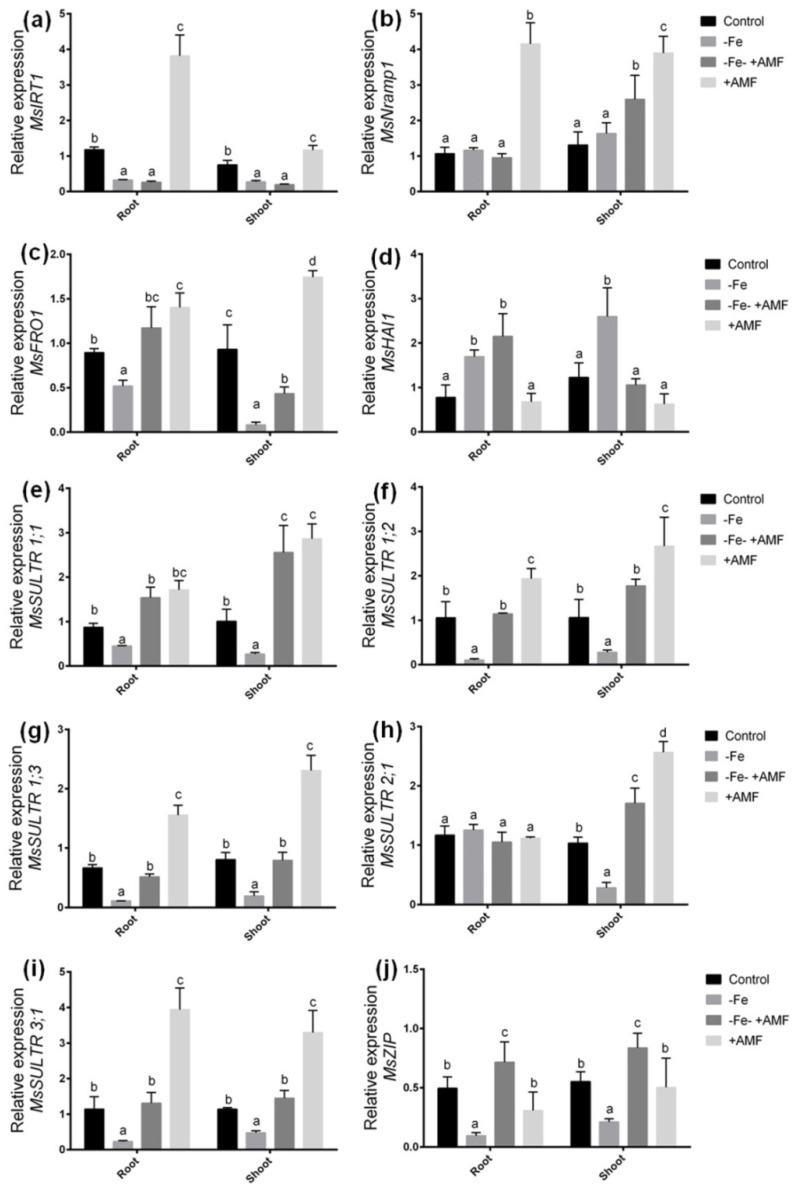
Quantitative expressions of candidate genes in alfalfa root and shoot in response to Fe deficiency with or without AMF. (**a**) Response of *MsIRT1*, (**b**) *MsNramp1*, (**c**) *MsFRO1*, (**d**) *MsHAI1*, (**e**) *MsSULTR1;1*, (**f**) *MsSULTR1;2*, (**g**) *MsSULTR1;3*, (**h**) *MsSULTR2;1*, (**i**) *MsSULTR 3;1*, and (**j**) *MsZIP* genes. The level of relative gene expression was analyzed using the dd^−∆Ct^ method, and *MsActin* was used as internal control. Data represent means ±SD (*n* = 3). Different letters above the error bar indicate significant difference at *p* < 0.05 level.

A quantitative real-time PCR (qRT-PCR) analysis was performed for the alfalfa plants cultivated with Fe deficiency and/or AMF supplementation with or without Fe deficiency to determine the expression of different transporter genes. In this study, *MsIRT1* showed significant down regulation in both root and shoot of alfalfa under Fe deficiency. Hence, the *MsIRT1* significantly up regulated in both root and shoot when plants were cultivated with AMF (Figure 5a). However, the expression of *MsNramp1* did not show any changes in root under Fe deficiency and/or AMF supplementation with Fe deficiency compared to controls (Figure 5b). Interestingly, plants cultivated with only AMF showed a significant upregulation in the expression of *MsIRT1* and *MsNramp1* in alfalfa compared to the other three treatments. The expression of *MsFRO1* gene significantly decreased due to Fe deficiency in both root and shoot compared to the controls (Figure 5c). However, alfalfa plants cultivated with AMF under Fe deficiency (−Fe+AMF) showed a significant increase in *MsFRO1* expression in comparison with Fe-deficient (−Fe) plants in both root and shoot. Plants grown with only AMF supplementation (+AMF) showed a significant increase in the expression of *MsFRO1* gene in either root or shoot compared to the rest of the treatments. Importantly, cellular H^+^-ATPase gene *MsHAI1* significantly expressed in root and shoot under Fe deficiency, though the expression was higher in root after supplementation of AMF (Figure 5d).

To validate the S data in ICP-MS analysis, we further studied the expression pattern of different S-transporter genes. In the present study, the expression of *MsSULTR1;1*, *MsSULTR1;2*, *MsSULTR1;3,* and *MsSULTR3;1* significantly decreased in root and shoot in response to Fe- deficiency compared to the controls. However, the addition of AMF with or without Fe-deficiency (+AMF, −Fe+AMF) caused a significant increase in the expression of *MsSULTR1;1*, *MsSULTR1;2*, and *MsSULTR1;3* in root and shoot compared to the plants grown under Fe-deficiency (Figure 5e–g). *MsSULTR2;1* gene showed no significant changes among the treatments in roots. However, Fe-deficiency (−Fe) resulted in a significant decrease in *MsSULTR2;1* expression in shoot following Fe-deficiency compared to the controls. The expression of *MsSULTR2;1* was higher in AMF supplementation (+AMF) compared to Fe-deficient conditions in the shoot (Figure 5h). *MsSULTR3;1* found to be higher in either root or shoot after AMF addition compared to the plants grown under Fe deficiency (Figure 5i). In addition, we checked the expression of Zn-transporter gene *MsZIP* to verify the ICP-MS results. The expression of *MsZIP* significantly decreased in both root and shoot under Fe-deficiency, and it was found to be increased after supplementation of AMF with or without Fe-deficiency (+AMF, −Fe+AMF). However, the expression of *MsZIP* was prominently higher in AMF supplementation with Fe-deficient condition (Figure 5j).

### 2.5. Role of AMF on Antioxidant Enzymes in Alfalfa

**Figure 6:**
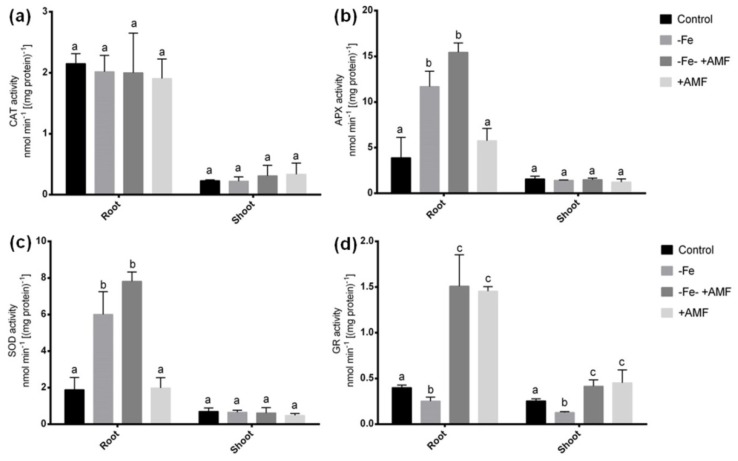
Effects of Fe deficiency and AMF on antioxidant enzymes activities in alfalfa root and shoot. (**a**) Changes of catalase (CAT), (**b**) ascorbate peroxidase (APX), (**c**) superoxide dismutase (SOD), and (**d**) glutathione reductase (GR) under Fe deficiency with or without AMF. Data represent means ± SD (*n* = 3). Different letters above the error bar indicate significant difference at *p* < 0.05 level.

Several antioxidant enzymes were determined to evaluate whether AMF does possess antioxidant defense to scavenge ROS damage induced by Fe deficiency in alfalfa. In this study, we did not see any significant changes in CAT activity in either root or shoot among the treatments (Figure 6a). APX and SOD activities significantly increase in roots of plants cultivated under Fe deficiency with or without AMF supplementation (−Fe, −Fe+AMF) in the culture compared to the non-treated controls (Figure 6b,c). Although these activities were comparably higher in root after the addition of AMF with Fe deficient condition. Plant cultivated solely with AMF showed a similar tendency of APX and SOD activities to that of controls in roots. In shoot, APX and SOD enzymes did not show any changes among the treatments. Further, Fe deficiency resulted in a significant decrease in GR activity in both root and shoot of alfalfa compared to non-treated controls. However, GR activity showed a remarkable increase in both root and shoot due to AMF supplementation with or without Fe deficiency (+AMF, −Fe+AMF) compared to the plants grown without AMF treatments (Figure 6d).

### 2.6. Effect of AMF on S-Metabolites

Although Fe deficiency showed no significant decrease in glutathione and cysteine concentration in roots of alfalfa compared to controls, AMF addition to Fe deficiency caused a significant increase in these two S-metabolites compared with the Fe-deficient and control plants (Table 2). Plant cultivated with AMF supplementation showed similar glutathione and cysteine concentration pattern in roots to those plants grown in control or Fe-deficient condition. Methionine showed no significant changes in the roots of alfalfa among the treatments (Table 2).

### 2.7. Sulfur Deprivation Effect Under Fe Deficiency

**Figure 7:**
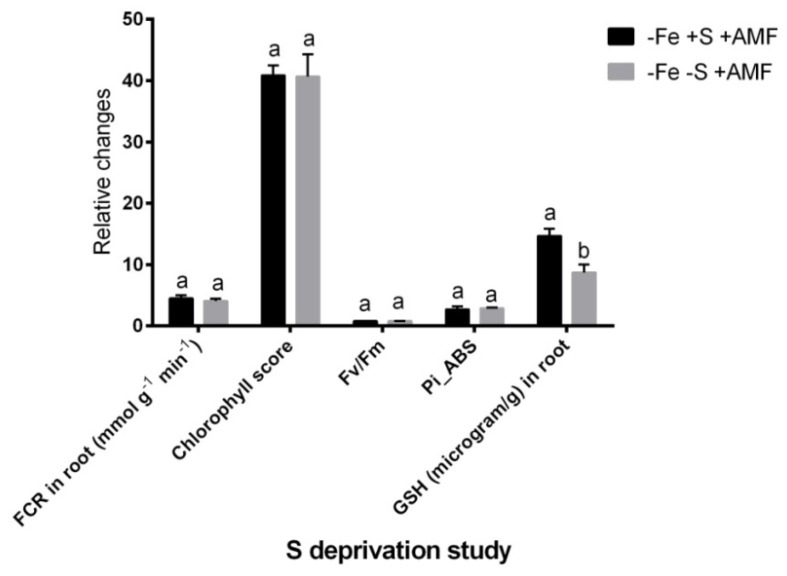
Sulfur deprivation study in alfalfa. Physiological and biochemical changes under Fe deficiency (−Fe) in the presence or absence of sulfur (+/−S) and AMF. In the figure, FCR, Fv/Fm, Pi_ABS, and GSH indicate the ferric chelate reductase, quantum efficiency of photosystem II, photosynthesis performance index, and glutathione accumulation with or without S supplementation in alfalfa, respectively. Data represent means ± SD (*n* = 3). Different letters above the error bar indicate significant difference at *p* < 0.05 level.

We measured a few key parameters in the absence and presence of S in the nutrient media to see whether S deprivation affects AMF-mediated mitigation of Fe-deficiency symptoms in alfalfa. In this study, alfalfa plants grown under Fe deficiency with S and AMF supplementation (−Fe+S+AMF), showed no significant changes in root FCR, SPAD score, Fv/Fm and Pi_ABS compared to the plants grown in similar growth conditions without S (Figure 7). However, S deprivation under Fe deficiency with AMF (−Fe-S+AMF) supplementation showed a significant decrease of glutathione concentration in alfalfa root compared to the Fe-deficient with S supply having AMF supplementation.

## 3. Discussion

Mitigation of mineral deficiency through plant-microbe interactions is highly demanding in plant production. In this study, the detrimental effects of Fe deficiency on alfalfa plants were obvious, with decreased biomass, photosynthesis inefficiency and increased ROS accumulation. These findings are in accordance with other legume plants vulnerable to Fe deficiency [6,26]. Although the induction of Strategy I strategies is often occurred in plants, genotypic variations in response to Fe-deficiency also exist in plants [3,11]. The role of AMF in diminishing Fe deficiency stress in plants is generally well known other than *Medicago* species. This study not only reveals the beneficial role of AMF but also describes the crosstalk among the Fe-deficiency mitigation, different biochemicals, and molecular responses in alfalfa.

### 3.1. Improvement of Alfalfa Plant Biomass and Physiological Parameters

Due to Fe deficiency, a shortage of Fe and nutritional imbalance are induced in plant cells. In this study, AMF significantly increased the dry mass and width of alfalfa under Fe deficiency. Apart from it, chlorophyll synthesis and photosynthesis parameters remarkably improved in leaves of alfalfa, suggesting that AMF supplementation restored the Fe balance in alfalfa plants even though plants were cultivated on Fe-deficient conditions. Fe deficiency changes the structure of thylakoid membrane resulted in a decline of PS-II efficiency in plants [27,28,29] and interferes chlorophyll biosynthetic pathway dependent on Fe containing enzymes [30,31]. Chlorophyll *a* fluorescence has been used as a powerful tool for studying photosynthetic systems in plants exposed to different abiotic stresses [32]. The leaf Fv/Fm ratio, considered a reliable diagnostic indicator of damage, significantly increased in mycorrhizal plants under Fe deficiency. Our study implies that maintenance of photosynthesis efficiency by providing sufficient Fe to the reaction center is independently governed by AMF regardless of the S status of the plants.

### 3.2. Enhancement of Mineral Content in Alfalfa

The effects of Fe deficiency on the alternations of different nutritional elements have been studied in several plants [10,33]. In this study, Fe deficiency resulted in a dramatic decrease in Fe along with Zn, S, and Ca in alfalfa plants. This suggests that Fe-deficiency induced retardation in alfalfa includes alternation in nutritional balance in the whole plants when plants are not efficient to increase the ability to acquire Fe through Strategy I mechanisms. In this study, AMF supplementation not only improved Fe concentration but also increased other minerals in alfalfa plants, suggesting that mycorrhizal association enables nutritional flow to the host plants, which is not mineral specific. Improvement of Fe uptake, which is the primary focus of this work, was further supported by the increased FCR activities in response to AMF under Fe deficiency. FCR enzyme activity is responsible to increase Fe availability at the root plasma membrane in Strategy-I plants [10]. AMF boosted Fe uptake because of the fungal hyphae and broader root area serving the host plant to acquire Fe under Fe deficiency [34]. This is possibly occurred by glomalin, a metal-sorbing protein surrounded by mycorrhizal hyphae [35,36].

As AMF improved Fe uptake in alfalfa plants, we primarily focused on the expression analysis of candidates’ genes associated with Fe uptake, transport, and bioavailability. In this present study, *MsIRT1* significantly down-regulated due to Fe deficiency but showed no changes when AMF was supplemented on Fe-deficient plants. However, it was induced in both and shoot when plants were only grown on AMF-supplemented media. It does suggest the *MsIRT1* does not improve Fe uptake under Fe deficiency but might be involved with enhanced Fe uptake when growth conditions are optimum in alfalfa plants. Another Fe transporter, *MsNramp1*, showed similar expression but showed slight induction due to the combination of Fe deficiency and AMF supplementation. In *Arabidopsis thaliana*, *IRT1* functions as is a primary transporter responsible for a broad range of metals under Fe deficiency [7]. *NRAMP1* also showed its efficiency as the pioneer regulated Fe deficiency responses and might play a critical role in Fe homeostasis in plants [37]. Interestingly, we found significant upregulation of *MsFRO1* responsible for FCR activity in either root or shoot, more predominantly in root due to AMF addition under Fe deficiency, suggesting that AMF plays an important role in Fe reductase and its bioavailability in alfalfa plants under Fe deficiency. *Glomalin*, a metal-sorbing protein surrounded by mycorrhizal hyphae potentially contributes to the availability and transferability of Fe transferability in the mycorrhizosphere [35,36]. Our findings suggest that the action of Fe(III)-chelate reductase ensures sufficient bioavailability of Fe at the plasma membrane, which is then normally absorbed into root cells by *MsIRT1* and *MsNramp1* plants following AMF supplementation under Fe deficiency in alfalfa plants. Since the presence of AMF symbiosis facilitates adequate bioavailable Fe in the rhizosphere, thus alfalfa plants did not suffer from Fe deficiency.

In a targeted study, we did not find any correlation of S affecting FCR activity under Fe deficiency following AMF supplementation in alfalfa plants. In tomato, S deficiency prevented the Fe-deficiency induced increase of FCR [38]. In this study, acidification in the rhizosphere due to proton (H^+^) secretion seems to be a common response observed in Strategy-I plant, although the magnitude was greater due to AMF in alfalfa. This evidence was supported by the upregulation of *MsHAI1* gene in roots, expressed more pronouncedly in the presence of AMF under Fe deficiency. In addition, H^+^-ATPase provides energy in the periarbuscular membrane resulted in improved nutrient exchange in plant cells [39]. 

The results from the ICP-MS analysis and previous reports [11,38] concerning the role of S on Fe-deficient plants, we extended gene expression analysis of range S transporters in alfalfa subjected Fe deficiency with or without AMF. We demonstrated that the drastic increase of S in either root or shoot was significantly increased following AMF, pinpointing that whether AMF induces S transporter genes and or influence Fe-uptake or other protective defense in alfalfa plants subjected to Fe deficiency. In this study, expression of *MsSULTR1;1*, *MsSULTR1;2*, *MsSULTR1;3*, *MsSULTR3;1*, and *MsZIP* significantly upregulated following AMF supports the hypothesis that AMF-mediated increased S and Zn accumulation is associated with the inductions of S and Z transporter genes in alfalfa plants. In barley, *HvZIP13* showed induction by mycorrhizal colonization following Zn deficiency [40]. In this study, Fe-deficiency induced interruptions in Zn, Ca, and P were also restored due to AMF supplementation. These elemental improvements may also assist alfalfa plants in coping with Fe deficiency. AMF is known to enhance Zn [41] and P [42] in *Medicago trunculata*. Taken together, AMF-mediated alleviation of Fe deficiency not only associated with the restoration of Fe status but also improve the increase of vital elements, such as Zn, Ca, and P in alfalfa.

### 3.3. Antioxidant Responses and Alleviation of Oxidative Stress Injury 

Plants suffer from an oxidative injury due to Fe deficiency. The onset of oxidative damages in plants is more prominent due to Fe deficiency as Fe is the central constituent or factor of major antioxidant enzymes [43,44]. However, the upregulation of antioxidant enzymes or metabolites to overcome elevated ROS is one of the characteristics of a Fe-efficient plant species/cultivar. In this present study, Fe-deficiency caused elevated APX and SOD activities in roots of alfalfa plants, suggesting its general responses to withstand oxidative burst. In the case of AMF supplementation under Fe deficiency, APX and SOD showed similar responses to that of Fe-deficient plants but additionally boosted up the activity of GR in both root and shoot after AMF supplementation with or without Fe deficiency. It does suggest that the rise of GR is associated with the presence of AMF in alfalfa plants under Fe deficiency. Although alfalfa plants were not efficient to induce GR activity, the addition of AMF facilitated its induction and thus, may play a role to scavenge ROS. GR is an important antioxidant that acts against H_2_O_2_ and O₂^−^ [45]. Further, this enzyme is essential for GSH, an *S*-metabolite that protects the cell from oxidative damage [46]. This phenomenon was also consistently supported the increased GSH and cysteine accumulation in alfalfa roots, as evident by our HPLC analysis due to AMF treatment under Fe deficiency. S deprivation study further suggests that the rise of GSH is only dependent on the availability of sufficient S in the cell. Taken together, AMF enables elevated S-metabolites under Fe deficiency, possibly by influencing ascorbate-glutathione cycles to scavenge ROS damage in alfalfa plants. The elevated antioxidant properties of mycorrhizal alfalfa plants may be associated with enhanced plant growth under Fe deficiency.

## 4. Materials and Methods

### 4.1. Alfalfa Cultivation and AMF Supplementation

Viable seeds of alfalfa (*Medicago sativa* L. cv. Vernal) were obtained from Kings Seeds, United Kindom. AMF inoculum consisted of 25% of each endomycorrhizal spore (*Glomus intraradices*, *Glomus mosseae*, *Glomus aggregatum*, *Glomus etunicatum*). Firstly, alfalfa seeds were surface sterilized with 70% ethanol for 5 min before geminated in germination tray supplemented with distilled water on tissue paper at room temperature. The germinated seeds were then transferred to the 500 g of sterile potting mixed (Gardener’s Supply Company, Burlington, VT, USA) substrate [1:1 (*v*/*v*), perlite: sand] supplemented with micro and macro-elements [20,47]. The chemicals were as follows (µM): KNO_3_ (16000), Ca(NO_3_)_2_.4H_2_O (6000), NH_4_H_2_PO_4_ (1000), MgSO_4_.7H_2_O (2000), KCl (50), H_3_BO_3_ (25), MnSO_4_.4H_2_O (2), Na_2_MoO_4_.2H_2_O (0.5), and CuSO_4_.5H_2_O (0.5). In addition to these, there four treatments: control (25µM Fe-EDTA); −Fe (1.0 µM Fe-EDTA); −Fe +AMF (1.0 µM Fe-EDTA and 250 mg AMF spore/pot) and AMF+ (25µM Fe-EDTA and 250 mg AMF spore/pot). The soil was irrigated with solution every 3 days. The plants were kept in the growth chamber at 25 °C having 60% relative humidity, 200 μmolm^−2^ s^−1^ light intensity, and long-day light conditions (14 h light/10 h dark). The plants were harvested 4 weeks after transferring to the potting mix (soil bulk density: 1.44 g/cm^3^) when the plants were in the vegetative stage.

### 4.2. Determination of Mycorrhizal Colonization

The mycorrhizal colonization was observed in alfalfa roots following the method described earlier [48]. Alfalfa roots were cut into 1 cm, followed by submerged with 10% (*w*/*v*) KOH at 90 °C for 2 h and then stained with trypan blue. The root sections were then mounted on slides with polyvinyl alcohol and glycerol followed by visualization in a digital microscope at 20× magnification.

### 4.3. Analysis of Morpho-Physiological Parameters

After 4 weeks in cultivation, root and shoot lengths were measured using a digital caliper. Afterward, root and shoot were dried in an oven for 72 h at 80 °C before measuring the dry weight. The chlorophyll content of young trifoliate leaves was measured by SPAD meter (Minolta, Japan) on intact plants growing in the growth chamber. Furthermore, FluorPen FP 100 (Photon Systems Instruments, Drasov, Czech Republic) was used to investigate the chlorophyll fluorescence characteristics, such as Fv/Fm (quantum efficiency of photosystem II) and Pi_ABS (photosynthesis performance index). Hence, alfalfa plants cultivated in the potting mix, plants were adapted at dark for 1 h prior to take reading.

### 4.4. ICP-MS Analysis

After completing of treatments, alfalfa root samples were washed three times with deionized water to remove surface AMF. Afterward, roots were incubated at 4 °C in 1st solution (10 mM MES) and 2nd solution (10 mM MES + 1 mM EDTA) followed by 2–3 times washing with deionized water. Alfalfa root and shoot were dried individually at 80 °C for 72 h. Equal amount of dried sample from each treated group was weighed and digested using a method described previously [49] with little modification. Root and shoot samples were digested using a digestion mixture (HNO_3_/HClO_4_, 3:1 *v*/*v*), and the targeted nutrients in the digestion solution were determined by inductively coupled plasma mass spectroscopy (ICP-MS, Agilent 7700), and multi-element ICP-standard-solution (ROTI^®^STAR, Roth, Germany) was used for preparing standard curves. Three independent replicate samples were considered for the analysis.

### 4.5. Determination of Fe Chelate Reductase Activity

The Fe (III)-FCR activity was measured using the described method [8]. Plant samples were rinsed with 0.2 mM CaSO_4_ and deionized water. Briefly, 100 mg of sample was homogenized in 1.5 mL tube using 100 mM Fe(III) EDTA, 0.10 mM MES-NaOH (pH 5.5), 300 mM ferrozine. The sample containing tubes was placed for incubation at 23 °C for 20 min in the dark. Finally, the absorbance of samples was read at 562 nm. The FCR activity was determined using the extinction co-efficient for ferrozine (M^−1^∙cm^−1^).

### 4.6. Estimation of Rhizosphere Acidification

For this study, Fe-deficiency was induced indirectly by adding 15 mM NaHCO_3_ (pH 7.5) to the solution culture [20,47] for measuring the secretion of H^+^ that may affect the pH of the rhizosphere [50]. Each plant was cultivated in 50 mL small container for 4 weeks under four different treatments mentioned previously. The initial and end pH of the solution was recorded by a digital pH meter. 

### 4.7. Determinatin of Soluble Protein

Total soluble protein in alfalfa samples was measured using bovine serum albumin (BSA) as standard [51]. Briefly, plant sample was ground with 50 mM Tris-HCl buffer (pH adjusted to 7.5), 2-mM EDTA, and 0.04% (*v*/*v*) β-mercaptoethanol. The samples were centrifuged at 12,000 rpm for 10 min. The clear supernatants were added with 1 mL Coomassie Brilliant Blue (CBB). Finally, the absorbance of the solution was measured at 595 nm.

### 4.8. Estimation of Electrolyte Leakage

The electrolyte leakage (EL), recommending the cytoplasmic membrane solidity, both in roots and shoots of alfalfa was measured by a digital electric conductivity meter [52]. Surface elements were eliminated through cleansing plant root and shoot with non-ionized water. Afterwards, the samples were kept in a pot having 20 mL of non-ionized water and shaken for two hours with incubation at the ambient temperature. Finally, the electrical conductivity of the solution was recorded.

### 4.9. Determination of Hydrogen Peroxide and Cell Death

Hydrogen peroxide (H_2_O_2_) accumulation was measured from the alfalfa sample using 0.1% trichloroacetic acid (TCA) according to the method described previously [53]. The extracted fluid was spun for 15 min at 10,000 rpm before the cellular remains were separated. The upper aqueous phase was added with KI (1 M) and KP-buffer (10 mM, pH adjusted to 7.0) and kept in the darkness for 60 min. Finally, the optical frequency of the distillate was read at 390 nm using a Shimadzu UV-1650PC spectrophotometer.

The number of cell death percentages (%) was determined, as described previously [54]. Shortly, 0.2 g of fresh tissue was stained with 2 mL Evan’s blue solution for 15 min. The suspension was subsequently treated with 1 mL of 80% ethanol for 10 min. The mixture containing tubes were placed in a water bath (Vision Scientific, Seoul, Korea), and incubated at 50 °C for 15 min, followed by rotated (12,000 rpm) for 10 min. The absorbance of supernatant read at 600 nm. Finally, total cell death in tissue was calculated on fresh weight and relative percentage (%) basis.

### 4.10. RNA Extraction, cDNA Synthesis, and qRT-PCR Analysis

Total RNA was extracted from the root and shoot of alfalfa plants using high quality RNeasy^®^ plant mini kit (QIAGEN, Hilden, Germany). Briefly, 100 mg of plant tissue was homogenized with RNA extraction buffer containing 1% (*v*/*v*) β-mercaptoethanol (β-ME), followed by centrifuged (≥ 12,000 rpm) for 2 min. RNA was recovered from the supernatant and final RNA yield was obtained by adding RNase-free water. A micro-volume UV/Vis spectrophotometer (UVISDrop-99, Avans Biotechnology Corp., Taipei, Taiwan) was used for measuring RNA concentration. Samples with RNA concentration of ≥ 200 ng/μL were selected for subsequent analysis. The first strand cDNA synthesis was performed with 1 μg of total RNA using the cDNA synthesis kit (Bio-Rad, Hercules, CA, USA). Quantitative real time polymerase chain reaction (qRT-PCR) was performed by the CFX96 Real Time System (Bio-Rad, Hercules, CA, USA) using iQ^TM^ SYBR^®^ Green Supermix (Bio-Rad, Hercules, CA, USA). The primers were designed based on *Medicago sativa* homologs available in NCBI/GenBank/EMBL databases (Supplementary Table S1). Total 20 μL reaction mixture consisted of 10 μL of iQ^TM^ SYBR^®^ Green Supermix, 1 μL of template cDNA, 1μL of forward primer (10 μM), 1μL of reverse primer (10 μM), 7 μL of dd H_2_O. The amplification was performed using the following programs: 95 °C for 3 min, followed by 40 cycles at 95 °C for 10 s, 60 °C for 30 s, where melt curve conditions were 55 °C to 95 °C with an increment of 0.5 °C for 5 sec. The levels of relative gene expression were analyzed using the dd^−∆Ct^ method [55], where the housekeeping gene *MsActin* used as an internal control.

### 4.11. Analysis of S-Metabolites

Cysteine, glutathione, and methionine were analyzed in roots by HPLC-technique using Empower3™ software (Waters Corporation, Milford, MA, USA). The chromatography system was connected to a C18 reverse phase-HPLC column (pore size: 300 A, particle size: 5μm, pH range: 1.5–10, dimension: 250 mm × 10 mm) was supplied with buffer A (0.1% TFA and water) and buffer B (0.1% TFA and 80% acetonitrile) at the gradient of 1–24 min 100% A, 25–34 min 100% B and 35–40 min 100% A as mobile phase. Prior to injection, the samples and standards were diluted (100×) and filtered using 0.22 μm Minisart Syringe Filters (Finetech, Taichung, Taiwan). S-metabolites were detected at 280 and 360 nm with a Waters 2489 dual absorbance detector [56].

### 4.12. Determination of Antioxidant Enzymes

In order to determine antioxidant enzymes, 100 mg plant tissue was homogenized with 100 mM potassium phosphate (KP-buffer, pH 7.0). The mixture was spun for ten minutes (8000 rpm), and the supernatant was separated in a new tube. To determine superoxide dismutase (SOD), the plant extracts (100 µL) combined with EDTA (0.1 mM), NaHCO_3_ (50 mM, pH 9.8) and epinephrine (0.6 mM) following the protocol used previously [57]. The confirmation of adrenochrome was recorded at 475 nm. For APX, extracts was mixed with 0.1 mM EDTA, 50 mM KP-buffer (pH 7.0), 0.1 mM H_2_O_2_, 0.5 mM ascorbic acid, and 0.1 mL extraction [58]. Subsequently, the absorbance of supernatant was measured at 290 nm and APX activity was calculated with an extinction co-efficient (2.8 mM^−1^ cm^−1^). The CAT reaction assay was performed using 100 mM KP-buffer (pH 7.0), 6% H_2_O_2_ and 100 µL plant extract, and absorbance was recorded at 240 nm (extinction co-efficient 0.036 mM^−1^ cm^−1^) at the 30s-60s interval. Further, 100 μL plant extract was mixed with 0.2 mol KP-buffer (pH 7.0), 1 mM EDTA, 20 mM oxidized glutathione (GSSG) and 0.2 mM NADPH for GR assay. The reaction was started with GSSG and declined in absorbance at 340 nm in the response of NADPH oxidation. The GR activities were ascertained utilizing the extinction co-efficient (6.12 mM^−1^ cm^−1^) [59].

### 4.13. Statistical Analysis

Experiments were performed using a randomized block design (RBD) with three independent biological replications for each sample. The significance of an individual group of data was evaluated at *p* ≤ 0.05 by analysis of variance (ANOVA) followed by Duncan’s Multiple Range Test (DMRT) or *t*-test, where applicable using the SPSS Statistics 20 system. In addition, graphical figures were prepared and analyzed using software GraphPad Prism (version 6.0).

## 5. Conclusions

This study provides new insight and the mechanistic basis of AMF-mediated Fe-deficiency mitigation in alfalfa. In this study, AMF showed a considerable improvement in Fe-deficiency induced plant biomass, photosynthesis efficiency, and cellular damages in alfalfa. Mycorrhizal fungi showed a substantial increase in Fe, Zn, S, Ca, and P concentrations in alfalfa plants under Fe deficiency. Moreover, the restoration of Fe was highly dependent on Fe availability and mobilization, mainly mediated by ferric chelate reductase and rhizosphere acidification enhanced by AMF supplementation under Fe deficiency. However, these strategy-I responses acted independently under mycorrhizal inoculation regardless of the S status of the plants. In addition, AMF provides an antioxidant defense to counteract Fe-deficiency induced oxidative injury mainly through the increase of S metabolites and GR activity in alfalfa. These findings provide an essential background for mitigating Fe deficiency retardation through fungal inoculation in legume plants.

## Figures and Tables

**Table 1:** ICP-MS analysis of elemental concentration (mg∙g^−1^ DW) in root and shoot of alfalfa cultivated under Fe deficiency (-Fe) with or without AMF. Data represent means ± SD (*n* = 3). Different letters above the tabulated values indicate significant difference at *p* < 0.05 level.

Root				
	Control	-Fe	-Fe +AMF	+AMF
Fe	149 ± 19.2 ^b^	88 ± 17.6 ^a^	131 ± 19.4 ^b^	177 ± 48.6 ^b^
Zn	147 ± 50.3 ^b^	52 ± 15.8 ^a^	123 ± 23.4 ^b^	112 ± 4.5 ^b^
S	14623 ± 323.6 ^b^	11107 ± 1662.5 ^a^	16177 ± 2571.4 ^b^	16856 ± 1637.3 ^b^
Ca	738 ± 220.9 ^b^	447 ± 88.8 ^a^	1282 ± 174.8 ^b^	1195 ± 60.0 ^b^
P	953 ± 145.1 ^c^	296 ± 55.1 ^a^	551 ± 77.6 ^b^	824 ± 125.5 ^c^
K	126 ± 64.0 ^a^	137 ± 24.9 ^a^	104 ± 21.6 ^a^	155 ± 55.8 ^a^
Mg	196 ± 21.2 ^a^	245 ± 56.6 ^a^	476 ± 69.2 ^b^	455 ± 11.2 ^b^
**Shoot**				
Fe	62 ± 10.6 ^b^	34 ± 6.5 ^a^	55 ± 13.2 ^b^	58 ± 7.7 ^b^
Zn	51 ± 7.6 ^b^	24 ± 1.4 ^a^	51 ± 9.6 ^b^	44 ± 10.8 ^b^
S	3538 ± 262.9 ^b^	2340 ± 66.4 ^a^	4479 ± 589.8 ^b^	4031 ± 1006.5 ^b^
Ca	460 ± 62.3 ^a^	428 ± 45.9 ^a^	751 ± 18.0 ^b^	658 ± 88.2 ^b^
P	260 ± 44.1^c^	78 ± 7.0 ^a^	176 ± 42.5 ^b^	292 ± 60.2 ^c^
K	47 ± 10.8 ^a^	47 ± 20.1 ^a^	53 ± 13.4 ^a^	68 ± 49.8 ^a^
Mg	39 ± 11.8 ^a^	35 ± 4.7 ^a^	40 ± 2.6 ^a^	32 ± 3.6 ^a^

**Table 2:** S-metabolites (µg g^−1^ FW) in root of alfalfa cultivated under Fe deficiency (−Fe) with or without AMF. Data represent means ± SD (*n* = 3). Different letters above the tabulated values indicate significant difference at *p* < 0.05 level.

S-metabolites	Control	−Fe	−Fe+AMF	+AMF
Glutathione	5.5 ± 1.09 ^a^	6.8 ± 0.57 ^a^	15.0 ± 1.76 ^b^	6.2 ± 0.59 ^a^
Cysteine	2.4 ± 0.84 ^a^	2.0 ± 0.96 ^a^	6.5 ± 1.43 ^b^	2.7 ± 0.19 ^a^
Methionine	4.3 ± 0.81 ^a^	4.9 ± 1.03 ^a^	4.7 ± 0.85 ^a^	4.4 ± 0.75 ^a^

## Supplementary Materials

Supplementary materials can be found at https://www.mdpi.com/1422-0067/21/6/2219/s1.
