# Supplementary material for: Arbuscular Mycorrhizal Symbiosis Mitigates Iron (Fe)-Deficiency Retardation in Alfalfa (Medicago sativa L.) Through the Enhancement of Fe Accumulation and Sulfur-Assisted Antioxidant Defense
Source: Int J Mol Sci. 2020 Mar 23;21(6):2219. doi: 10.3390/ijms21062219 (PMC7139841; doi:10.3390/ijms21062219)
Supplement: Supplementary file 1 [file ijms-21-02219-s001.pdf]

Supplementary Figure S1. Screening of colonization with fungal hyphae and vesicle following with or without Arbuscular mycorrhizal fungi (AMF) supplementation.

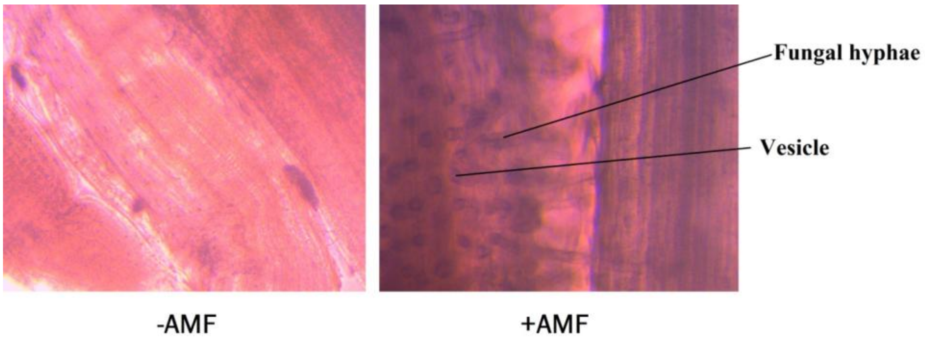

Supplementary Table S1. List of primers used for qRT-PCR

Gene	Primer	Sequence
MsActin	Forward	ACCGGTGTGATGGTTGGTAT
	Reverse	GCCACACGAAGCTCATTGTA
MsSULTR1.1	Forward	TTCGGATGCGTAATAGGT
	Reverse	TTGCAATCCACAGGAACAAA
MsSULTR1;2	Forward	CAGATAAGCAAGGAGTAGAA
	Reverse	TCATAGAACCAACGACAT
MsSULTR1;3	Forward	ATTTATGCCGTCATGGGTAG
	Reverse	TTGGGATCAATCTCATTCTGTA
MsSULTR2;1	Forward	AAATGTTGTCAATGGGAATC
	Reverse	CCAGGAAGTGCGGAGA
MsSULTR3;1	Forward	CCACCATCACTCACAGA
	Reverse	ATAATGCCAACAATAAGG
MsIRT1	Forward	TTTACCCTTGGCGACACGTT
	Reverse	CATGAACCCGGTCCCAAGAA
MsNramp1	Forward	GCATTGCTAGCCTCAGGACA
	Reverse	TCCATGCGTATGCAGGTGAT
MsFRO1	Forward	GGTGACACGTGGATCATCTG
	Reverse	TTGCAATCCACAGGAACAAA
MsHAI1	Forward	GGCAGGAGTCATTTGGTTGT
	Reverse	TGCTTCTCTGCAAACCCTTT
MsZIP	Forward	ATGGGAATCGCATTGCTAAG
	Reverse	CTGCGGTTTGAAGCCTTTAG
